# Outcomes of off- and on-hours admission in ST-segment elevation myocardial infarction patients undergoing primary percutaneous coronary intervention: A retrospective observational cohort study

**DOI:** 10.1097/MD.0000000000004093

**Published:** 2016-07-08

**Authors:** Jin Geng, Xiao Ye, Chen Liu, Jun Xie, Jianzhou Chen, Biao Xu, Bingjian Wang

**Affiliations:** a Department of Cardiology, Drum Tower Hospital, Nanjing University Medical School, Nanjing; b Department of Cardiology, Huai’an First People's Hospital, Nanjing Medical University, Huai’an; c Department of Endocrinology, Zhejiang Provincial People's Hospital, Hangzhou; d Department of Cardiology, Yangzhou No.1 People's hospital, Yangzhou, China.

**Keywords:** mortality, off-hours admission, primary percutaneous coronary intervention, ST-segment elevation myocardial infarction

## Abstract

Studies evaluating the outcomes of patients with ST-segment elevation myocardial infarction (STEMI) undergoing primary percutaneous coronary intervention (PCI) are scarce, particularly in China. The purpose of present study was therefore to compare the impact of off-hours and on-hours admission on clinical outcomes in STEMI patients from China.

We retrospectively analyzed 1594 patients from 4 hospitals. Of these, 903 patients (56.65%) were admitted during off-hours (weekdays from 18:00 to 08:00, weekends and holidays) and 691 (43.35%) were during on-hours (weekdays from 08:00 to 18:00).

Patients admitted during off-hours had higher thrombolysis in myocardial infarction risk score (4.67 ± 2.27 vs 4.39 ± 2.10, *P* = 0.012) and longer door-to-balloon time (72 [50–96] vs 64 [42–92] minutes, *P* < 0.001) than those admitted during on-hours. Off-hours admission had no association with in-hospital (unadjusted odds ratio 2.069, 95% confidence interval [CI] 0.956–4.480, *P* = 0.060) and long-term mortality (unadjusted hazards ratio [HR] 1.469, 95%CI 0.993–2.173, *P* = 0.054), even after adjustment for confounders. However, long-term outcomes, the composite of deaths and other adverse events, differed between groups with an unadjusted HR of 1.327 (95%CI, 1.102–1.599, *P* = 0.003), which remained significant in regression models. In a subgroup analysis, off-hours admission was associated with higher long-term mortality in the high-risk subgroup (unadjusted HR 1.965, 95%CI 1.103–3.512, *P* = 0.042), but not in low- and moderate-risk subgroups.

This study showed no association between off-hours admission and in-hospital and long-term mortality. Stratified analysis indicated that off-hours admission was significantly associated with long-term mortality in the high-risk subgroup.

## 1 Introduction

Patients with acute myocardial infarction (AMI) admitted during off-hours have a higher mortality than those admitted during on-hours, which may be attributed to the decreased staff numbers and levels.^[1–6]^ However, this conclusion becomes controversial because of inconsistent results from numbers of studies.^[7–12]^ Kostis et al^[1]^ reported that 30-day mortality was significantly higher for AMI patients admitted on weekends than on weekdays, but this disparity disappeared when invasive procedures was forced into the regression model. Similarly, Kumar et al^[4]^ also demonstrated a declined mortality risk on weekends admission than on weekdays after additional adjustment for cardiac catheterization. These suggested that lower availability of cardiac intervention might lead to the difference in mortality between off-hours and on-hours groups. Patients admitted during off-hours were less likely to undergo invasive procedures,^[1,2,11]^ such as percutaneous coronary intervention (PCI), an accepted management for AMI. In previous studies, however, only a few AMI patients (5–60%) underwent primary PCI.^[1,3,11,12]^ Moreover, outcomes for off-hours and on-hours varied among countries,^[13]^ and most above-mentioned studies were performed in western countries.^[1,2,4,7–12]^ Whether off-hours hospital presentation is associated with higher risk of short- and long-term outcomes in China remains unclear.

In addition, there are few studies reporting the effect of off-hours admission according to the severity of ST-segment elevation myocardial infarction (STEMI). Recently, a study evaluated the effect of weekend admission on in-hospital mortality according to Killip classification and indicated that the effect became more significant from Killip I to IV subgroups.^[3]^ The thrombolysis in myocardial infarction (TIMI) risk score is a clinical tool for predicting the short- and long-term mortality in patients presenting with STEMI,^[14,15]^ which contains the term of Killip class. To our knowledge, no study has assessed the effect of off-hours admission in STEMI patients with different TIMI risk score.

Therefore, the aims of present study were to compare the short- and long-term outcomes between STEMI patients undergoing primary PCI admitted during off-hours and those admitted during on-hours, and to determine how the effect changed according to the TIMI risk score.

## 2 Methods

We retrospectively included all STEMI patients undergoing primary PCI from January 2012 to December 2015 in 4 grade A tertiary hospitals. The diagnosis of STEMI was based on the recent guideline.^[16]^ Exclusion criteria included fibrinolytic therapy before admission and life expectancy <2 years. The eligible patients were divided into off-hours and on-hours groups according to the admission time. Off-hours were defined as weekdays from 18:00 to 08:00, weekends and holidays. On-hours were defined as weekdays from 08:00 to 18:00. During off-hours, an on-call team was contacted upon acceptance of an STEMI patient for primary PCI. All on-call team members would be in the hospital within 15 to 30 minutes. During on-hours, the on-call team was in the hospital and can perform primary PCI as soon as possible. This study was approved by the ethics committee at each participating hospital. All patients involved signed the informed consent.

Baseline variables included demographic, bloods and clinical data, medical history, invasive procedure characteristics, and length of hospitalization. Bloods data, such as hemoglobin, glucose, and creatinine were tested before primary PCI, whereas serum lipid levels were usually tested after primary PCI, because we routinely tested it at the second day after admission when patients were fasting. All patients received echocardiography measurement within 3 days after admission. Left ventricular ejection fraction (LVEF) and left ventricular end-diastolic diameter (LVEDd) were recorded for further analysis. Both day of admission and day of discharge were included in the length of hospital stay. The TIMI risk score contains 8 terms: age, history of diabetes, hypertension or angina pectoris, systolic blood pressure (BP), heart rate, Killip classification, weight, anterior ST-elevation or left bundle branch block and time to treatment, and ranges from 0 to 14 points.^[14]^ In this study, systolic blood pressure and heart rate were measured and recorded when patients arrived. Time to treatment was defined as symptom-to-balloon time.

Follow-up was performed from the admission until death or Jan 2016. The primary outcomes were in-hospital and long-term all-cause mortality. The secondary outcomes were in-hospital events, including death, reinfarction, recurrent ischemic symptoms and heart failure during hospitalization, and long-term major adverse cardiac events (MACE), defined as death, reinfarction and unplanned hospitalization. Death and reinfarction during hospitalization were included in long-term MACE. Any readmission due to cardiac reasons, such as angina pectoris, heart failure, and arrhythmia despite optimized pharmacological therapy, was defined as unplanned hospitalization. Follow-up data were obtained from at least 1 of the following 3 methods: medical records, telephone contact, and outpatient visitation.

Continuous data were presented as mean ± standard deviation or medians and interquartile range according to the results of Kolmogorov–Smirnov test. Categorical data were presented as numbers and percentages. Intergroup analyses were achieved using Student's *t* test for normally distributed continuous variables and the Mann–Whitney *U* test for non-normally distributed variables.^[17]^ The chi-square test or Fisher's exact test was employed for categorical variables. The effect of off-hours on in-hospital events was evaluated by multiple logistic regression analysis and presented as odds ratios (ORs) and 95% confidence intervals (CIs). For the association of off-time admission and long-term MACE, Kaplan–Meier analysis and log-rank test were first used. Hazard ratios (HRs) and 95%CIs were calculated using Cox proportional hazards regression analysis. Two regression models were used: model 1 adjusted for variables which showed *P* < 0.1 based on univariate analyses,^[2]^ including sex, hypertension, previous MI, previous PCI/ coronary artery bypass graft (CABG), previous angina pectoris, current smokers, anterior MI, LVEF, LVEDD, and door-to-balloon (DTB) >90; model 2 additionally adjusted for known cofounders for mortality,^[7]^ including age, creatinine, systolic BP, heart rate, symptom-to-door time, diabetes mellitus, hemoglobin, glucose, TIMI flow 2 to 3 after PCI, and multivessel disease. Furthermore, patients were additionally divided into 3 subgroups according to the TIMI risk score: high (8–14 points), moderate (4–7 points), and low (0–3 points) risk groups. We conducted Kaplan–Meier survival curves and used log-rank test to compare the differences between off-hours and on-hours groups in each subgroups. SPSS version 22.0 (IBM SPSS, Armonk, NY) was used for our analyses. Statistical significance was considered when *P* < 0.05 (2-tailed).

## 3 Results

Of 1594 patients included in this study, 903 patients admitted during off-hours (56.65%) and 691 during on-hours (43.35%). Baseline characteristics were showed in Table 1. The off-hours group was more often male (81.28% vs 76.99%, *P* = 0.036) and had a high rate of anterior MI (48.06% vs 41.69%, *P* = 0.011) compared with the on-hours group. Lower LVEF (45.66 ± 6.72% vs 46.44 ± 5.99%, *P* = 0.017) and larger LVEDd (5.42 ± 0.40 vs 5.38 ± 0.42 cm, *P* = 0.045) were found in patients admitted during off-hours than in patients admitted during on-hours. Patients in the off-hours group had higher proportions of hypertension, previous MI, previous PCI/CABG, previous angina pectoris, and current smokers. We used the TIMI risk score to evaluate the severity of STEMI and found that off-hours admission tended to be associated with higher TIMI risk score compared with on-hours admission (4.67 ± 2.27 vs 4.39 ± 2.10, *P* = 0.012). For other variables, no statistical significance was found in univariate analyses (Table 1).

**Table 1:**
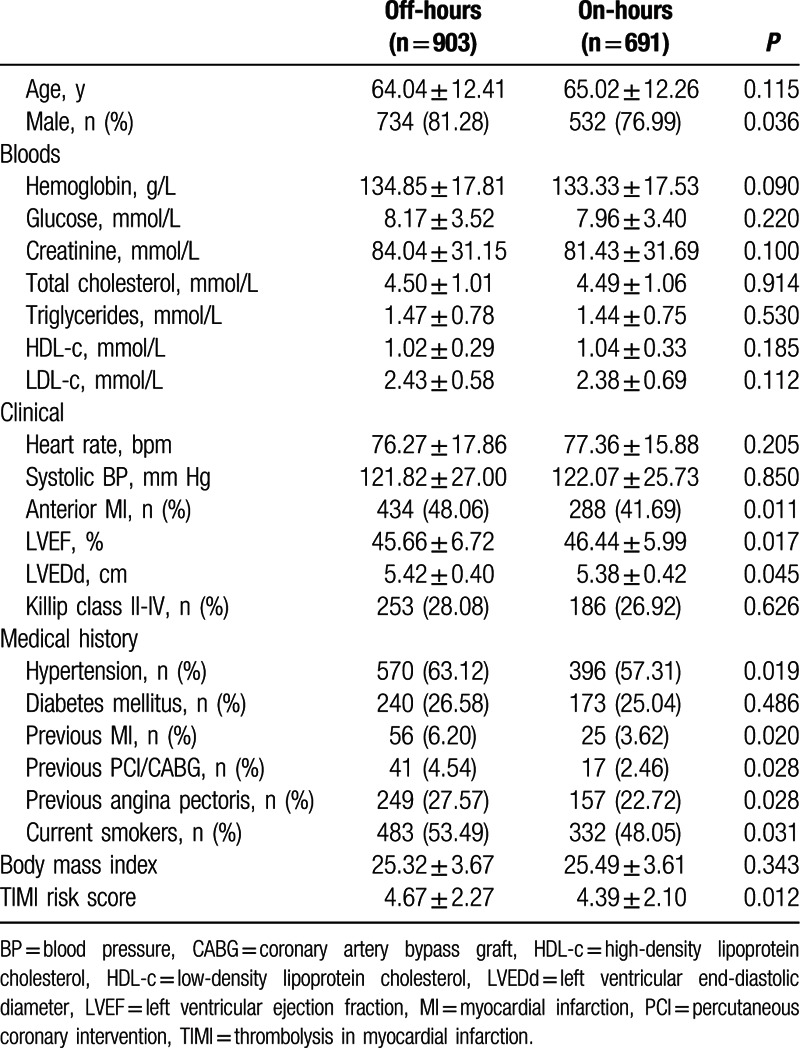
Baseline characteristics of off-hours and on-hours groups.

Table 2 showed invasive procedure characteristics and intervals. The infarct-related artery was more likely to be left anterior descending artery (47.51%) in the off-hours group, whereas in the on-hours group, 44.14% of the culprit artery was the right coronary artery and 40.96% was left anterior descending artery. Percentages of multivessel disease, ≥2 stents implanted and TIMI flow 2 to 3 before and after PCI did not differ between the 2 groups. Symptom-to-door time, symptom-to-balloon time, and procedure time were also similar between groups. However, patients admitted during off-hours had significant longer DTB time (72 [50–96] vs 64 [42–92] minutes, *P* < 0.001). There was no significant difference for hospital length of stay in these 2 groups (Table 2).

**Table 2:**
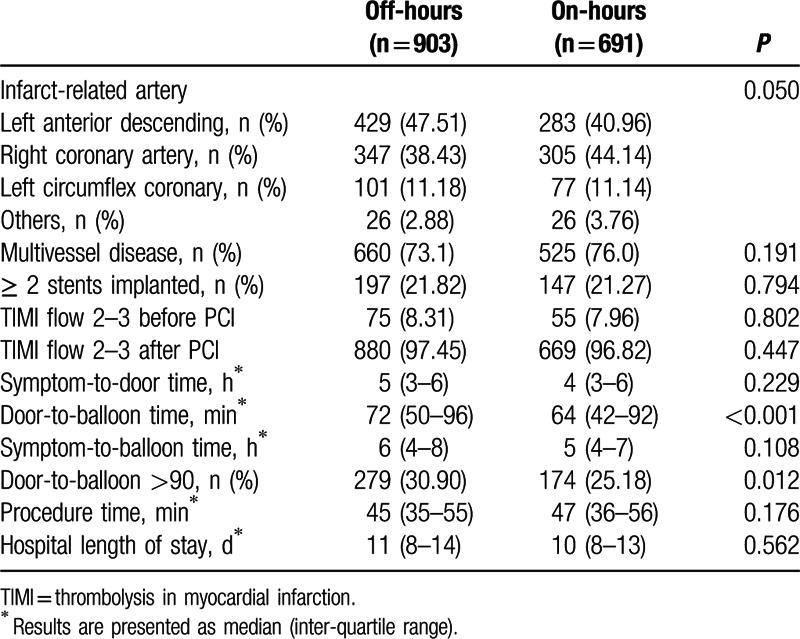
Procedure characteristics and intervals of off-hours and on-hours groups.

During a median hospital stay of 10 days, there were 24 deaths (2.66%) in the off-hours group and 9 deaths (1.30%) in the on-hours group. No significant difference was found in in-hospital mortality between groups (unadjusted OR 2.069 [0.956–4.480], *P* = 0.060). Two regression models were used for further adjusted analyses; adjusted ORs were 1.724 (95%CI, 0.914–4.521, *P* = 0.078) for model 1 and 1.835 (95%CI, 0.823–4.642, *P* = 0.112) for model 2. Moreover, the rates of reinfarction, recurrent ischemic symptoms, and heart failure were similar in patients admitted during off-hours compared with those admitted during on-hours. In the logistic regression models, there was also no significant difference between the 2 groups (Table 3).

**Table 3:**
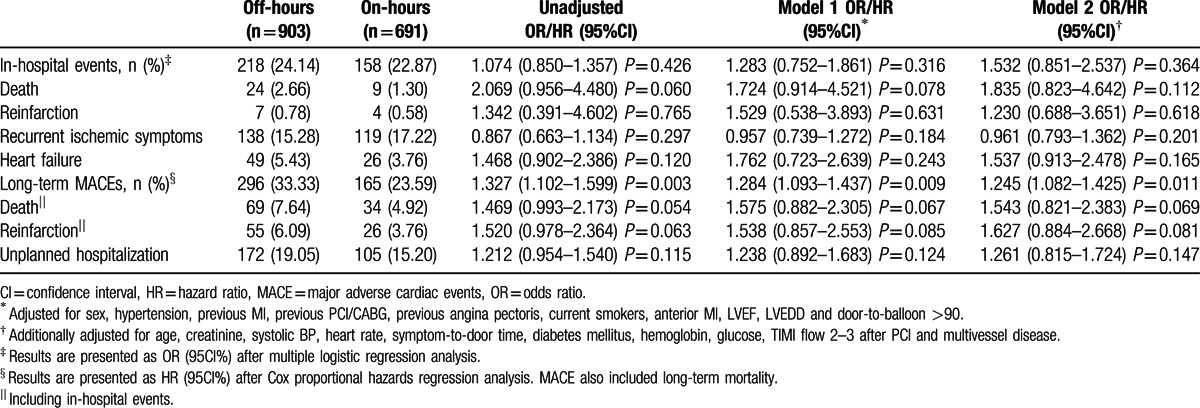
In-hospital and long-term outcomes of off-hours and on-hours groups.

The median follow-up period was 16 (8–29) months in the off-hours group and 16 (7–28) months in the on-hours group (*P* = 0.272). During this period, 103 STEMI patients undergoing primary PCI died (6.46%), of which 69 were in the off-hours group and 34 in the on-hours group. Unadjusted HR was 1.469 (95%CI, 0.993–2.173, *P* = 0.054) for off-hour admission. Besides, off-hour admission had no effect on reinfarction and unplanned hospitalization during follow-up (unadjusted HRs: 1.520, 95%CI, 0.978–2.364, *P* = 0.063; 1.212, 95%CI, 0.954–1.540, *P* = 0.115, respectively). In the multiple Cox proportional hazards models, there was also no significance difference in long-term mortality, reinfarction, and unplanned hospitalization between groups (Table 3). However, long-term MACEs, the composite of deaths, reinfarctions, and unplanned hospitalization, differed between groups with an unadjusted HR of 1.327 (95%CI, 1.102–1.599, *P* = 0.003). In the multiple Cox proportional hazards model, off-hours admission remained significantly associated with higher rates of MACEs (HRs: 1.284, 95%CI, 1.093–1.437, *P* = 0.009 for model 1 and 1.245, 95%CI, 1.082–1.425, *P* = 0.011 for model 2).

**Figure 1:**
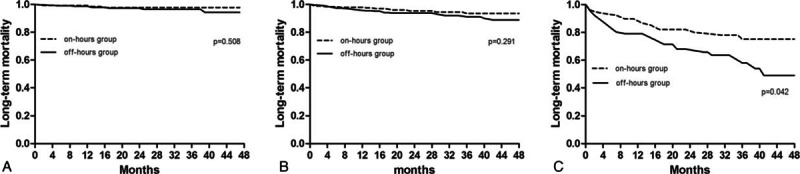
(A) Kaplan–Meier curves for long-term mortality in the low-risk subgroup. (B) Kaplan–Meier curves for long-term mortality in the moderate-risk subgroup. (C) Kaplan–Meier curves for long-term mortality in the high-risk subgroup.

In addition, we repeated the analyses of long-term mortality in 3 subgroups and results were showed in Table 4. Off-hours admission had no effect on long-term mortality in the low-risk subgroup (unadjusted HR 1.456, 95%CI 0.488–4.429, *P* = 0.508) and in the moderate-risk subgroup (unadjusted HR 1.499, 0.818–2.748, *P* = 0.291). In the high-risk subgroup, however, it was significantly associated with mortality during long-term follow-up (unadjusted HR 1.965, 95%CI 1.103–3.512, *P* = 0.042). When adjusted for confounders in the Cox models 1 and 2, statistical significance was still existed (Table 4). Kaplan–Meier survival curves of long-term mortality in these 3 subgroups were illustrated in Fig. 1.

**Table 4:**
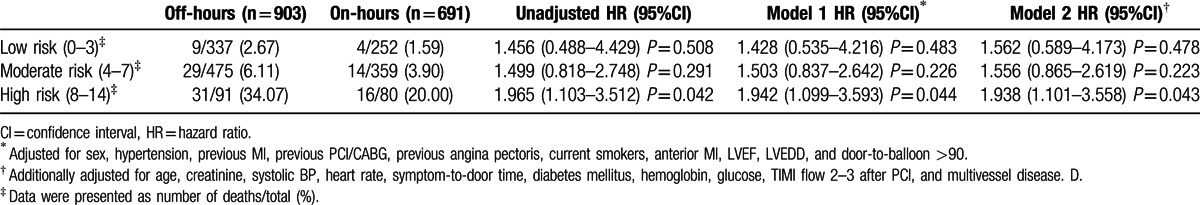
Stratified analyses for long-term mortality according to the TIMI risk score.

## 4 Discussion

Our analysis of 1594 STEMI patients with primary PCI indicated that patients admitted during off-hours had similar in-hospital and long-term mortality, and also the in-hospital events. However, presentation during off-hours was associated with a high risk of developing MACEs, even after adjustment for 2 regression models. When patients were divided into 3 subgroups according to the TIMI risk score, it seemed that off-hours admission had an adverse effect on long-term mortality in the high-risk subgroup, but not in the other 2 groups.

The results of previous studies comparing the effect of off-hours and on-hours admission in AMI patients on clinical outcomes were inconsistent.^[1,3–5,9,11,12]^ Recently, a meta-analysis enrolled 48 studies and concluded that off-hours admission was associated with higher short-term mortality in patients with AMI.^[18]^ However, high heterogeneity was found among the included studies, and subgroup analyses showed different results in some cohort, suggesting that enrollment of STEMI and non-STEMI patients, various definition of off-hours admission and even the different region where patients were included might cause the inconsistent results. Moreover, previous studies had suggested that reduced use of PCI might partially explain the higher mortality in AMI patients admitted during off-hours.^[1,4]^ Therefore, only the patients diagnosed with STEMI and received primary PCI therapy were included in present study.

In the recent years, 3 studies from Western countries were conducted to determine whether the clinical outcomes differed when the timing of primary PCI or arrival changed.^[7,8,10]^ In the year of 2012, Noman reported no differences in in-hospital and long-term mortality in STEMI patients from United Kingdom.^[7]^ Similarly, a registry study from United Kingdom also concluded no differences in short- and long-term outcomes between off-hours and on-hours groups.^[10]^ In both studies, patients were divided into either off-hours or on-hours group based on the timing of primary PCI performed. Patients from a prospective cohort trial who were stratified into the off-hours group according to the arrival time seemed to experience similar clinical outcomes as those stratified into the on-hours group.^[8]^ It seems that STEMI patients undergoing primary PCI have similar outcomes regardless of the timing of admission in current western countries. However, it might not be appropriate to generalize these results in China, a developing country.

Chinese people are more likely to endure the pain of diseases. Some STEMI patients with less severe symptoms may delay seeing a doctor during off-hours, whereas patients with more severe symptoms would be hospitalized. Therefore, STEMI patients admitted during off-hours may have longer symptom-to-door time and may be have more severe symptoms than those admitted during on-hours. In the present study, the median symptom-to-door time was 5 for off-hours admission and 4 for on-hours admission, but there reached no statistical significance. We used the TIMI risk score to evaluate severity of STEMI and found patients in the off-hours group were with higher risk score compared with those in the on-hours group, supporting our previous hypothesis. An earlier multicenter registry study recruited 11,516 STEMI patients from 1994 to 2002 in Germany showed a higher TIMI risk score and a longer DTB time in patients admitted during off-hours,^[19]^ which was similar to our results. Another study from Japan also reported more patients admitted during off-hours were with high Killip class.^[3]^ However, <70% of patients underwent primary PCI in these 2 studies, which might lead to the higher in-hospital mortality in STEMI patients.^[20]^ In contrast, all STEMI patients underwent primary PCI in our cohort and in-hospital and long-term mortality had no significant difference between groups in both univariate and multiple regression analyses with *P*-values approximate to 0.05. However, long-term MACEs, the combined endpoint of long-term mortality, reinfarction and unplanned hospitalization, were significantly higher during off-hours compared with on-hours, suggesting that failure to determine the difference in mortality might owe to lack of power. If more STEMI patients were included, there might be statistical significance.

Based on our results, here are some reasons for the high risk of MECEs in the off-hours group. First, STEMI patients admitted during off-hours were more severe with high TIMI risk score and subsequently decreased left ventricular function. Second, worse clinical outcomes in the off-hours group might be due to a potential longer symptom-to-door time in our cohort, although no statistical difference was found between groups. Third, patients admitted during off-hours often waited for an on-call team and took longer time from arrival to revascularization, as reported in previous studies.^[8,11,18,19]^ However, off-hours admission remained significantly associated with higher rates of MACEs after multivariate regression analysis. STEMI patients may be have a relative 20% to 40% increased short- and long-term mortality owing to an ∼30 minutes delay in DTB time,^[21–23]^ whereas a difference of DTB time< 30 minutes have shown no influence on clinical outcomes.^[8,24,25]^ In the present study, the median difference of DTB time was only 12 minutes, which might explain for the results of multiple analysis. Additionally, our stratified showed that off-hours admission was associated with higher long-term mortality in the high-risk subgroup, but not in low- and moderate-risk subgroups, indicating that decreased hospital staffing during off-hours might lead to possible reduction in optimal treatment,^[1,3]^ especially for the STEMI patients with more severe symptoms. Because more severe STEMI patients might require many staff for prompt care, whereas less severe patients might not. The insufficient staff might be another reason for failure to detect the influence of the important baseline characteristics on the regression results. To reduce the unfavorable effect of off-hours admission, an intervention, including expanded diagnostic services, improved discharge processes and increased physician management during off-hours might be a good choice.^[26]^

To our knowledge, this is the first study to assess the association of off-hours admission with short- and long-term clinical outcomes in primary PCI-treated STEMI patients from China. Besides, we used detailed clinical, bloods, medical history, and procedure data to evaluate the associations, which was different from previous studies.^[1,2,4,6–9]^ We also provided that STEMI patients admitted during off-hours were more severe, which might be one of the reasons for unfavorable prognosis in STEMI patients admitted during off-hours. On the other hand, the present study has some limitations. First, this is a retrospective observational study with inability to avoid selection and confounding bias. There were some imbalances of the baseline characteristics. However, randomization of enrolled patients was not feasible in an observational cohort design, and we used regression models to adjust these confounders. Second, there might be lack of power to detect the difference in mortality for a relatively small sample size. Third, our results might not be generalized for the whole Chinese population, because STEMI patients in the present study were only from 4 hospitals located in East China. Moreover, there are only about 30% STEMI patients undergoing primary PCI in China.^[27]^ Finally, for the inherent limitation of retrospective study, we had no detailed medicine records of included patients after discharge, which might influence the long-term mortality and MACEs. There is a need of large study with high quality to cover the whole Chinese population and to detect the small differences between groups.

## 5 Conclusions

STEMI patients undergoing primary PCI admitted during off-hours had no differences in in-hospital and long-term mortality compared with those admitted during on-hours, even after adjustment for confounding factors. However, off-hours admission showed significant association with higher risk of MACEs, suggesting the lack of power to detect the difference in mortality between groups. In a subgroup analysis, off-hours admission was associated with higher long-term mortality in the high-risk subgroup, but not in low- and moderate-risk subgroups.
