# Outcomes of off- and on-hours admission in ST-segment elevation myocardial infarction patients undergoing primary percutaneous coronary intervention: A retrospective observational cohort study: Erratum

**DOI:** 10.1097/01.md.0000494755.80250.91

**Published:** 2016-09-09

In the article “Outcomes of off- and on-hours admission in ST-segment elevation myocardial infarction patients undergoing primary percutaneous coronary intervention: A retrospective observational cohort study”,^1^ which appeared in Volume 95, Issue 27 of *Medicine*, Dr. Geng's affiliation was originally given incorrectly. The article has since been corrected online.
